# Nurse Migration in Australia, Germany, and the UK: A Rapid Evidence Assessment of Empirical Research Involving Migrant Nurses

**DOI:** 10.1177/15271544221102964

**Published:** 2022-06-23

**Authors:** Jamie B Smith, Doreen Herinek, Robyn Woodward-Kron, Michael Ewers

**Affiliations:** 1 Institute of Health and Nursing Science, Charité – Universitätsmedizin Berlin, Corporate Member of Freie Universität Berlin and Humboldt-Universität zu Berlin, Berlin, Germany; 2 85084 The University of Melbourne Faculty of Medicine Dentistry and Health Sciences, Melbourne, Australia

**Keywords:** migration, policy, empirical research, UK, Germany, Australia

## Abstract

Forecasts predict a growing shortage of skilled nursing staff in countries worldwide. Nurse migration is already a common strategy used to address nursing workforce needs. Germany, the UK, and Australia are reviewed here as examples of destination countries for nurse migrants. Agreements exist between countries to facilitate nurse migration; however, it is not evident how nurse migrants have contributed to data on which these arrangements are based. We examined existing primary research on nurse migration, including educational needs and initiatives to support policymakers’, stakeholders’, and health professions educators’ decisions on measures for ethical and sustainable nurse migration. We conducted a rapid evidence assessment to review available empirical research data which involved, was developed with, or considered migrant nurses to address the research question: what are the findings of research that directly involves migrant nurses in producing primary research data? A total of 56 papers were included. Four main themes were identified in this research data: Research does not clearly define what is meant by the term migrant nurses; discrimination is often reported by migrant nurses; language and communication competencies are important; and structured integration programs are highly valued by migrant nurses and destination healthcare employers.

Migrant nurses continue to experience discrimination and reduced career opportunities and therefore should be included in research about them to better inform policy. Structured integration programs can improve the experience of migrant nurses by providing language support (if necessary), a country-specific bridging program and help with organisational hurdles. Not only researching migrant nurses but making them active partners in research is of great importance for successful, ethical, and sustainable migration policies. A broader evidence base, especially with regard to the views and experiences of migrant nurses and their educational support needs, should be promoted to make future immigration policy more needs-based, sustainable and ethically acceptable.

## Introduction

Globally, it is increasingly difficult for healthcare systems to recruit sufficient qualified nurses to meet their growing healthcare needs. The gaps in nursing workforces can be broadly attributed to two categories: factors that decrease the number of nurses in the workforce and factors that increase the demand for nurses. One of the factors adversely impacting replacement needs of the nursing workforce is the retirement of qualified nurses. This rate is predicted to increase over the next decade (World Health Organisation, 2020b). In addition, many nurses leave the profession in the context of the economisation of healthcare as demands for more productivity with fewer incentives leave them physically and mentally exhausted (Kordes et al., 2020). The additional need for qualified nurses in healthcare systems is largely due to the growing needs of ageing and chronically ill populations (OECD, 2020; World Health Organisation, 2020a).

High-income countries often look to international nurse recruitment to address their domestic nursing shortages (WHO, 2020b). These countries attempt to create mutually beneficial economic and political arrangements with lower-income countries to encourage nurses to migrate from one country to another (Connell & Brown, 2004). In some cases, these bilateral labour agreements (BLAs) aim to address labour rights, mutual recognition of qualifications and integration into destination countries (van de Pas & Mans, 2018). BLAs often contain feedback mechanisms to report comprehensively on the nurse migration process; however, in most cases, these are not well enacted (van de Pas & Mans, 2018). The formation of policy and the experiences of migrant nurses should be feedback to each other. For example, the BLA between Germany and the Philippines includes robust protections and involvement for migrant nurses; yet these protections have still not been implemented despite being ratified in 2015. While there are good intentions to include stakeholders in policy development and migration strategies, lack of involvement of migrant nurses and insufficient reporting mechanisms means there is little knowledge about nurses’ experiences of the migration process (Kordes et al., 2020; van de Pas & Mans, 2018).

We approach this area of research and policy as an interdisciplinary group of researchers with a public health, nursing, education and communication science background from Australia, Germany, and the UK. We are working collaboratively on a project focusing on the educational aspects of nurse migration in a global health context. In this context it should be acknowledged that the political landscape of the countries of interest here is changing significantly with regard to nurse migration policy, e.g., with the UK leaving the European Union and new staffing and nurse staffing laws in Germany and Australia. It is not transparent what role migrating nurses themselves play in the policy development process and to what extent their perspectives are taken into account in policy decisions. It is also unclear what research data on nurse migration that includes migrant nurses underpins these decisions. We have opted for a Rapid Evidence Assessment to provide an opportune overview of the situation.

## Aim

The aim of this research is to explore how migrant nurses are represented in published academic literature on this topic. We aim to collate, analyse, and assess this data to identify any gaps in research and to further support policymakers, stakeholders, and health professions educators in these three countries and internationally to make decisions that support ethical and sustainable nurse migration. This research is conducted as a rapid evidence assessment (REA), as described by Grant and Booth (2009), of academic literature on nurse migration in Australia, Germany, and the UK. The specific focus of this REA is empirical research that actively involves nurses who have migrated to one of these three countries. An REA was chosen to situate the available primary research in the social and political contexts of those countries.

## Background

Based on WHO estimations, in 2018 there was a global deficit of 5.9 million qualified nurses (WHO, 2020a). Given the differences between countries, nurses are not further categorised in this report, however, globally professionalised nurses are around 69% of the workforce, associate professionalised nurses around 22% and around 9% of nurses are not classified either way (see also Appendix A). Forecasts predict the deficit of nursing staff to continue to grow over the next decade, which also affects the countries under review here (Marć et al., 2019). By 2030, an additional 520,000 full-time nursing positions will be required in Germany alone to care for the ageing population (WHO, 2020a). This prediction is echoed in the UK and Australia in a manner proportional to their demographics (Buchan et al., 2019). Further characterisation of these global trends using OECD data is available in Appendix A.

One response to these nursing shortages is workforce migration. Globally, one in eight qualified nurses works in a different country than where they were educated as a nurse (WHO, 2020a). However, nurse migration creates a complex system as it can both contribute to nursing shortages in countries of origin and address them in destination countries. The direction of nurse migration is often influenced by nurses seeking better wages and working conditions (Squires & Amico, 2014). Economies of low- and middle-income countries often rely on remittances from international nurse migration (Connell & Brown, 2004; van de Pas & Mans, 2018). Countries such as Australia (Aiken et al., 2004), Germany (Kordes et al., 2020) and the UK (Buchan et al., 2019) typically organise their economic and skilled migration policies around these labour markets.

Until recently, Germany had not looked to migration as a possible solution for recruiting qualified nursing staff (Kordes et al., 2020). In addition to a general change in immigration policy due to demographic and economic circumstances, some authors argue that this situation changed with the introduction of minimum staffing levels in hospitals and in long-term care institutions in Germany. This created a new context for nurse staffing in Germany and more opportunities for employers to look internationally for qualified nurses (Zander-Jentsch & Busse, 2018). Kordes et al. (2020) identified four key areas in understanding nurse migration in Germany: 1) the legitimisation of migration as a solution to nurse staffing shortages; 2) the categorisation of migrants by their background and linguistic abilities; 3) the delegation of international recruitment to third-party private agencies; and 4) the emergent technologies of state and legislative processes that guide migration. Levels of complexity are also added to the context of German nurse migration when negotiating international nurse migration between priorities for the European Union and Germany as an individual member state.

In contrast, the UK has a long-standing history of health workforce immigration. The UK's National Health Service (NHS) has been employing overseas healthcare professionals, including qualified nurses, since it was established in 1948. In the early years of the NHS, both trained and trainee nurses, particularly from Ireland and Caribbean countries, were targeted for recruitment (Winkelmann-Gleed & Hakesley-Brown, 2006). However, patterns of overseas nurse migration to the UK have fluctuated over time. The variability in the total number of overseas nurses entering the UK over the years depends primarily on how many nurses are needed and how many nursing jobs are available in the UK (Buchan & Seccombe, 2013). The existing demand for nurses and the impact of the UK's exit from the European Union are likely to continue to make nurse migration an area of interest for the UK.

Similarly, Australia has a long migration history of nurse migration for many decades to address nursing shortages and that the source countries for qualified nurses have varied over time. The Australian census analysis, published by WHO in 2014 reports that the main sources of nurses migrating to Australia between 2006–2011 were India (3,697), Philippines (3704), followed by the UK (2,885), China (1,266), and North Africa/Middle East (946) (Hawthorne, 2014). This general source country trend remains accurate for the period since, with India and the Philippines by far the dominant sources.

The increasing diversity of source countries of nurse migrants means that the way in which nurses are educated is also more varied, which has significance for the assimilation of nurses into health systems in destination countries (Hawthorne, 2005). Nevertheless, to meet the increased demand for qualified nurses, the state has recently intensified its activities in the area of international recruitment. The Australian National Disability Insurance Scheme (NDIS) of 2013, which was fully implanted in 2020 (AHPRA, 2017), has increased the country's need for qualified nurses.

International migration of nurses affects not only the individuals who migrate but also impacts effective service provision, health policies, workforce planning, training and education, and social and economic development in both the destination and source countries. Therefore, nurse migration must become an integral consideration for various policy fields from development to labour markets and health and social to education policies (Buchan & Seccombe, 2013). In 2010, the World Health Assembly adopted the first WHO Code of Practice on the International Recruitment of Healthcare Personnel. The associated strategic development goals make it clear that migration should be managed in an ethical way and benefit both the destination countries and countries of origin (WHO, 2010). This code of practice also encourages policymakers in higher-income countries to focus on domestic education, further education, and social integration of migrant nurses. These initiatives need a coherent and sustainable evidence base that is supported by research and includes the perspectives of nurses with a migrant background (van de Pas & Mans, 2018).

## Methodology

We explored the research question: How do migrant nurses contribute to primary research data? Although there is a large body of statistics, secondary data, and policy statements on nurses’ migration, it is not clear what the perspectives and experiences of migrant nurses are and how these are represented in policy. The research aimed to identify common themes emerging from findings of empirical research literature regarding the views and experiences of nurses who migrated to Australia, Germany, and the UK, which should be considered in the design of migration policies. These countries were selected because they represent high-income countries with a strong interest in nurse migration but nevertheless rely on different approaches. In this research, which was conducted in July 2020 and updated in September 2021, we intended to capture a broad overview of the available literature on this topic.

### Research Method

#### Initial Scoping

searches of databases and online search engines were completed using the search terms nurs* OR pfleg* [translation: nursing] OR altenpfleg* [translation: geriatric nurse] OR krankenschwester OR krankenpfleg* [translation: nurse] AND migra* OR ausland OR ausländ* [translation: foreign country] OR oversea* OR foreign OR international* in combination with the three countries (Australia OR Germany OR United Kingdom OR UK).

#### Context

International economic databases (e.g., Eurostat and OECD) were assessed for contextual relevance.

#### Research Question Development

How migrant nurses are represented in participation, design or co-design in primary research was unclear in the background information, scoping review, and contextual data.

#### Method Selection

A Rapid Evidence Assessment (REA) following the model offered by Haby et al. (2016) fits appropriately the breadth of the research question, the resources, and the timeframe available.

#### Types of Data

Empirical research studies were sought, with priority given to sources with robust methodologies that closely adhere to the CONSORT (Antes, 2010) reporting guidelines for critically appraising quantitative research articles and published in either English or German. Data were also sourced from grey literature and data published by governmental organisations and used as signposts to potential primary research data; however, these data were not included in extraction. All data included satisfied the standard for reporting qualitative research or CONSORT reporting guidelines and the full text be available.

#### Types of Participants

Sources must produce empirical research that involves migrant nurses in the research process as participants or the research design examines the role of migrant nurses in Australia, Germany, and the UK. We consider nurse migrants and nurse migration as nurses working in a country different from where they were educated or in a country to which they moved to undertake nurse education and subsequently work as a nurse (van de Pas & Mans, 2018).

#### Types of Articles/Interventions

Studies that examine nurse migrants as participants, the role of migrant nurses or migrant nurses as co-designers and used peer-reviewed primary research methodologies.

#### Types of Comparisons and Outcome Measures

How migrant nurses are considered or represented in empirical research in Australia, Germany, and the UK.

#### Search Strategy for Identification of Studies

A comprehensive search of six databases (CINAHL, the Cochrane Library, EconLit, EMBASE, Health Systems Evidence and PubMed (MEDLINE) and two websites (Google and Google Scholar) were conducted including a reference search.

#### Grey Literature and Manual Search

Google, Google Scholar, CINAHL and PubMed (MEDLINE) include grey, unpublished and conference materials. These were included in the review to look for signposting to research not identified elsewhere.

#### Search Strategy

Searches of full texts were conducted using the keywords in the PerSPECTiF scheme (Booth et al., 2019): These keywords were selected in English and German to represent terms that may relate to nurses and nurse migrants in the three countries. No time limit was applied to the publication of the articles.

#### Domain of PerSPECTiF Scheme and Search Terms

nurs* OR pfleg* OR altenpfleg* OR krankenschwester OR krankenpfleg* OR international* AND migra* OR ausland OR ausländ* OR oversea* OR foreign AND Australia OR Germany OR Deutschland OR UK OR United Kingdom. (Table 1)

**Table 1.** Inclusion and Exclusion Criteria.

Criteria	Inclusion	Exclusion
Perspective	Migrant nurse or involving migrant nurses	Research that did not directly include migrant nurses
Setting	Working in a healthcare setting or nursing role	Not working in healthcare or a nursing role
Phenomenon of interest	Migrant nurses	Not migrant nurses
Environment	Australia, Germany or the UK	Other countries
Comparison	Inclusion of migrant nurses	Migrant nurses not reported as directed included in data production
Timing	No time limit	
Findings	Peer-reviewed empirical data. Published in English or German language.	Not peer-reviewed data. Grey literature, policy documents.

#### Screening and Selection of Data

One reviewer from the UK (JS) conducted the initial search screening according to the selection criteria. The reviewer had a low threshold for including potential papers (Haby et al., 2016) that could later be screened out. The full texts of sources initially selected for inclusion were then retrieved and screened again with the inclusion criteria. A second reviewer from Germany (DH) screened the excluded papers to identify any sources that may be relevant and should have been included. Disagreements were resolved by a discussion between the reviewers.

#### Data Extraction

**Figure 1.**
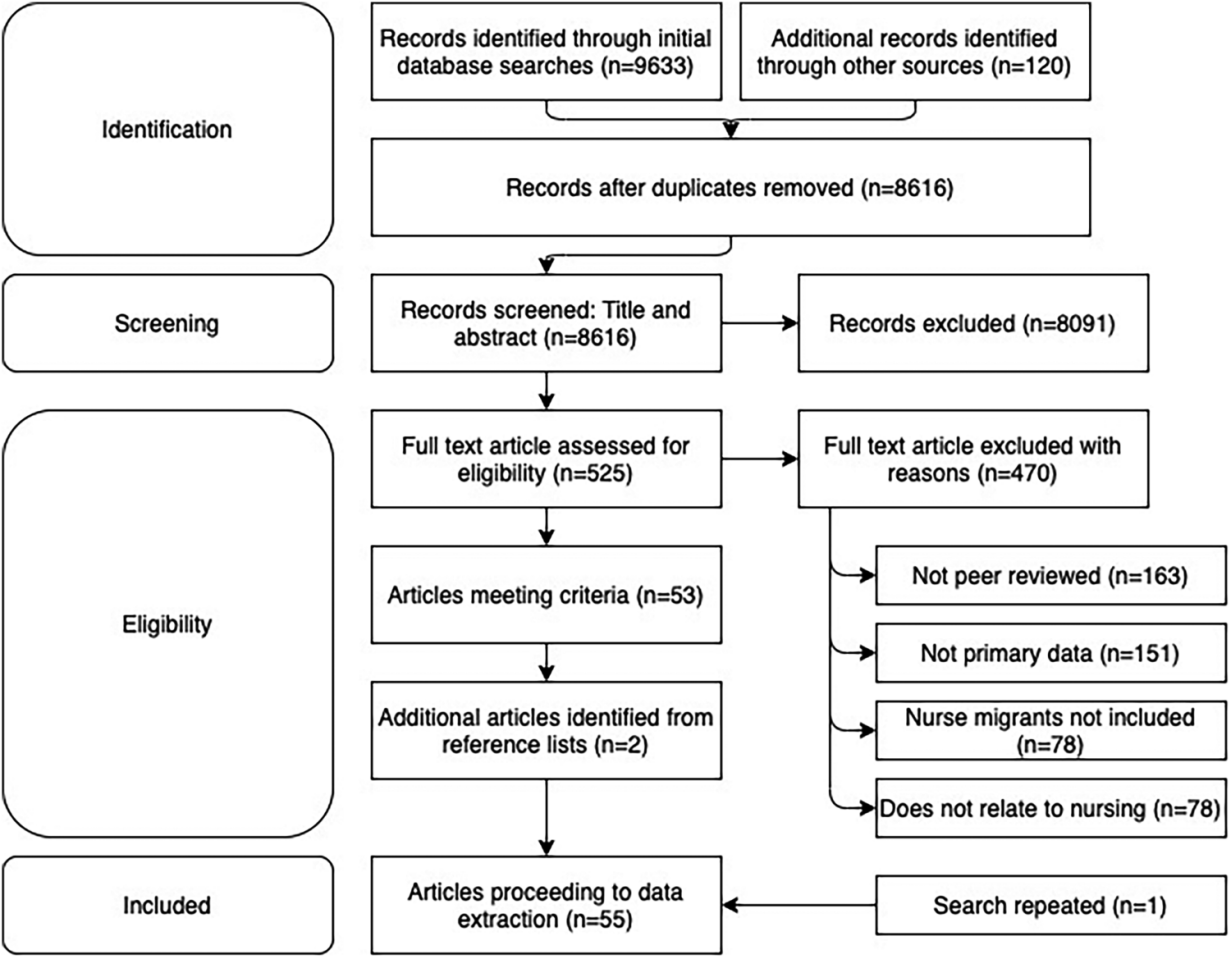
Search strategy and stages.

Search results from each database were downloaded into Endnote referencing software. Resources that met the inclusion criteria were extracted for full text (Figure 1).

#### Assessment of Methodological Quality

Only texts in English and German were included for analysis based on the countries included in the study and the language skills of the researchers. Quality assessment of all the texts used a quality assessment matrix adapted from CONSORT and Standards for Reporting Qualitative Research guidelines, which included a quality and bias assessment of literature.

#### Data Analysis

The adapted quality assessment framework was synthesised into a narrative report. This approach allows other data available to be mapped in the assessment of available evidence. Data analysis followed an inductive approach and aimed at, firstly, mapping the terrain of primary research involving migrant nurses and secondly, a qualitative description of the identified patterns (Gibbs, 2018). Data analysis followed generic qualitative data analysis processes by the four authors starting to independently re-read included articles, writing notes on insights and ideas, developing descriptive codes, reflecting on codes, developing categories, and eventually developing themes (Flick, 2018; Gibbs, 2018). This process eventually enabled the authors to develop the four themes as discussed below.

## Findings

56 research studies were found that met the defined selection criteria (*N* = 56). Empirical research data for the experience and views of migrant nurses exists in greater numbers in the Australian (*n* = 24) and British (*n* = 27) contexts than in published literature from Germany (*n* = 5). Qualitative research design is the most common approach in the published primary research. The number of participants varied across papers from 2 to 816. A summary data extraction table is presented in Appendix B. Table 2 below summarises the study designs of research included in this review.

**Table 2.** Research Methods Used in Extracted Literature.

	Australia	Germany	UK
Qualitative n =	17	3	26
Quantitative n =	5	1	0
Mixed n =	3	1	0
Total n =	25	5	26

OECD data which further contextualise this body of literature are available in Appendix A.

### Data Synthesis

The results of the data are summarised by four themes: (1) how migrant nurses are described in the literature; (2) language background and language competence; (3) discrimination; and (4) integration and structured educational programmes.

### Describing Migrant Nurses

Migrant nurses are most commonly described as Overseas Qualified Nurses (OQN) (Aggar et al., 2020; Alexis & Vydelingum, 2009; Ohr et al., 2016; Philip et al., 2019; Vafeas & Hendricks, 2018; Walters, 2008) or, alternatively and more recently, Internationally Qualified Nurses (IQN) (Aggar et al., 2020; Allan & Westwood, 2016; Deegan & Simkin, 2010; O’Callaghan et al., 2018); however, these terms are only used in Australian and UK literature. An abbreviation referenced only in some Australian texts is ‘Culturally and Linguistically Diverse’ (CaLD) (Crawford et al., 2016; Holmes & Grech, 2015; Salamonson et al., 2007) to distinguish between CaLD and English speaking background nurses. None of these differentiated terms are used in the German literature. Instead, legal terms are preferred that refer to a nationality other than German like ‘Ausland’ (foreign country) (Kumpf et al., 2016) or ‘Ausländer’ (foreigner) (Lauxen & Blattert, 2021). ‘Migrant’ is used to describe the research population across the three bodies of work (Al-Hamdan et al., 2015; Cecchin, 1998; Hardill & Macdonald, 2000; Schilgen et al., 2019).

Most of the literature included in this review using these definitions and names do not include people who may have migrated to Australia, Germany or the UK and then been educated as a qualified nurse. This is with the exception of one Australian study (Salamonson et al., 2007) that researched the experiences of nursing students to assess attitudes towards acceptance and awareness of nursing undergraduates from culturally diverse backgrounds.

### Language Background and Language Competence

Language proficiency in the first language of the destination country is required for nurses to practice. All research papers retrieved that were conducted in Germany acknowledge that the participants do not speak German as a first language. This is in contrast to the UK and Australian contexts, where the native language of the participants or their fluency in English is reported less often (Omeri & Atkins, 2002; Philip et al., 2019; Salamonson et al., 2007; Walters, 2008). However, this is noteworthy as all studies highlight communication proficiency as an important aspect of nurse migration.

Although 63% of nurses migrating to Australia come from countries where English is the first language (OECD, 2020 see Appendix A), this was acknowledged in only four research studies in the design or discussion (Crawford et al., 2016; Omeri & Atkins, 2002; Tie et al., 2019; Vafeas & Hendricks, 2018). One Australian study suggests that cultural assimilation into clinical practice can be difficult for nurses who speak English as their first language but who are not from this country (Tie et al., 2019). Differences in work cultures and expectations are cited as the rationale. Nevertheless, multiple other researchers reported that increased fluency in the language of the receiving country is associated with better assimilation and experiences for nurses and the health systems in which they work (Brunero et al., 2008; Kumpf et al., 2016; Sedgwick & Garner, 2017).

### Discrimination

Discriminatory practices were reported in the research data from all three countries, with similar experiences reported across the three countries. For example, migrant nurses reported feeling vulnerable (Crawford et al., 2016) in their new workplaces through mechanisms of alienation (Omeri & Atkins, 2002) and exclusion (Hardill & Macdonald, 2010; Kumpf et al., 2016; Tie et al., 2019).

Migrant nurses can feel alienated from their new teams, which is mediated through feelings of fear or lack of belonging (Walters, 2008). In the literature reviewed, these feelings of disempowerment are primarily attributed to insufficient language skills (Holmes & Grech, 2015; Kumpf et al., 2016; Stuart, 2012). The added pressures of learning and mastering a new language while simultaneously learning a new system can cause stress for nurse migrants (Schilgen et al., 2019). The evidence also suggests that some migrant nurses may experience discrimination from patients, other nurses and from service users (Alexis & Vydelingum, 2009; Lauxen et al., 2019).

Other stressors for nurse migrants can sometimes be due to structural and legislative restrictions in destination countries. This is recorded across data from Australia (Brunero et al., 2008), Germany (Schilgen et al., 2020) and the UK (Stuart, 2012). Migrant workers sometimes perceive training courses for skills and development as disproportionately difficult to access due to a perceived inequity because of their migration background (Kumpf et al., 2016). In addition, some national or state institutions may not recognise nursing qualifications achieved outside of the destination country (Walters, 2008). This can mean that migrants must work in a different, and often less respected position in the destination country. For example, migrant healthcare workers who have trained as a nurse outside of the UK may not be able to have their qualification recognised without further study and, therefore, choose to work as a healthcare assistant. In the UK, these jobs are often in community and long-term care settings (Stuart, 2012), which offer lower wages and status than other healthcare roles. Nurses report that their transferable skills are undervalued in these settings, leaving them frustrated and disempowered (Kumpf et al., 2016).

The underlying causes for implicit, explicit, and structural discrimination are not fully explored in the body of research included in this review; however, the multiple sources suggest they are complex and exist in multiplicities of contradictions between their labour, languages, and places of work (e.g. Hardill & Macdonald, 2010; Kumpf et al., 2016; Tie et al., 2019). There is also not yet an analysis of the different approaches to combating discrimination in the destination countries. One source recommends that there is a relation between the experiences of the migrant nurses and investments in social and cultural integration programmes (Aggar et al., 2020).

### Integration and Structured Educational Programmes

Based on the studies reviewed here, the most useful features of local structured integration programmes are those that focus on communication, cultural synthesis and clinical integration (Aggar et al., 2020; Sedgwick & Garner, 2017). These programmes are also described as engaging multiple stakeholders across several sectors, e. g. local hospitals, nurse regulators, universities, and politicians. Crawford et al. (2016) suggests some content for such local integration programmes, including cultural awareness and clinical communication, and report staff integration and communication as essential for patient safety. Structured integration programmes for migrating nurses are reported in the literature from the UK and Australia, but none from Germany. Hawthorne (2005) describes the significant investment in bridging programmes in Australia and a health policy shift since 1980 to provide these programmes for international nurses. In 2019, a ‘bridging programme’ for Overseas Qualified Nurses was established by a local higher education institution in Australia (Aggar et al., 2020), but there is little evaluation of the structure, goals, or content of this integration programme beyond their participants’ reports of usefulness. In the UK, a study also reported the importance of bridging programmes for migrant nurses to improve integration into health systems (Stubbs, 2017). Despite the frequent discussion of the importance of bridging programmes, little empirical research regarding these bridging programmes involving migrant nurses was found during this review.

First, there are specific educational needs of migrant nurses in a new clinical context. The needs of these nurses are different from those of domestically educated nurses (Aggar et al., 2020; Brunero et al., 2008) because they are competent and registered nurses in the country where they were educated. This positions them differently pedagogically from novice nurses, or nurses from the destination country returning to practice (Crawford et al., 2016) because they have a level of skill and expertise that they wish to develop in a different cultural context. Interviews with nurse educators responsible for teaching recent nurse migrants to Australia (Cecchin, 1998) reported that the educators did not feel prepared enough and that the teaching cultures they experienced with this group of nurses were very different to how they usually practice because they have clinical experience as qualified nurses in their countries of origin. The specific learning needs for migrant nurses is reported elsewhere as others call for specific educational frameworks for educators of overseas nurses (Allan & Westwood, 2016) and for distinct syllabi for such integration programmes (Aggar et al., 2020; Crawford et al., 2016).

Second, this data suggests that structured integration programmes improve the health and well-being of nurses, which in turn contributes to better recruitment and retention (Ohr et al., 2016). These programmes increase feelings of well-being and belonging alongside the camaraderie and support of colleagues and new friends (Tie et al., 2019). An increased perception of health and well-being enhances success rates and improves nurses’ experience and integration within an unfamiliar healthcare system. Feelings of discrimination can often be reduced or moderated by supportive colleagues (Schilgen et al., 2020) or by the development of strong friendships in destination countries (Vafeas & Hendricks, 2018). All studies report that nurses perceive the extent to which they are able to integrate as fundamental to their health and well-being. This accompanies multiple findings in the UK and Australia that structured integration programmes are essential to support positive outcomes from nurse migration (Aggar et al., 2020; Al-Hamdan et al., 2015).

## Discussion

Australia and the UK have longer histories of nurse migration than Germany, and they have far more established structures for conducting research on this issue in which migrant nurses are involved as active partners. However, even in these countries, much of the available peer-reviewed data comes from smaller qualitative studies with limited scope. There are more empirical research data for nurse migration available from Australia and the UK than Germany. While Australia and the UK have more published data available, the themes produced by all three countries are consistent: (1) how migrant nurses are described in the identified literature, (2) discrimination, (3) the importance placed on communication skills, and (4) the value of structured integration education programmes. It is not clear how these findings are used to support nurse migration policies and initiatives. BLAs such as the one discussed earlier between Germany and the Philippines (van de Pas & Mans, 2018) have mechanism built into them to include migrant nurses in the processes, however, are reportedly not well enacted. These findings suggest that these feedback mechanisms should be strengthened to improve the experiences of migrant nurses. These lessons can be incorporated in future discussions of policy, in a global context, as nurse migration is an international issue. Discrimination is the most prominent theme throughout the research included in this review; therefore, there is an ethical imperative to report this finding to the broadest possible audience.

The Australian literature included in this review appears to over-represent migrant nurses in research who do not speak English as their first language, despite 64% of OQN's being from countries with English as the first language (OECD, 2020). This representation of OQN's language ability may be due to changing trends in source and destinations countries of migrant nurses. Studies calling for migrant nurse participants may be unclear as to who is the target participant group. Nevertheless, this finding raises questions of why this is not addressed more directly.

Comparisons of nurse migrants and their experiences should consider that the terminology used to describe ‘nurse migrants’ varies in different international contexts, which has consequences for the way the topic is discussed. Australia and the UK use terminology that is sensitive to the different educational backgrounds of nurse migrants (like OQN, IQN) or to aspects that can be addressed in integration programmes (culture, language). In contrast, Germany uses terminology that tends to derive from citizenship law and does not take educational aspects into account. Furthermore, the different titles of nurses or caregivers may create inaccurate comparisons. The terms, ‘nurse’ ‘registered nurse’ and ‘migrant nurse’ have different meanings across the three countries discussed. These differences have implications for future research when drawing upon an international research base for policy and project design and must be considered to ensure appropriate comparisons are made.

In all three countries, there is a gap in empirical research when it comes to sufficiently considering the perspectives and experiences of migrant nurses in planning, conducting, and publishing on this topic. This gap in knowledge is made more relevant by each country's identification of nurse migration as a potential solution for increasing nurse shortages. Despite the established WHO ethical principles for an international approach to managed nurse migration, nurse migrants have not been considered in this policy process through research and suggests the need for a more participatory approach when dealing with nurse migration.

A point emphasised is the contrast between the available primary and secondary data. This difference is characterised by fewer quantitative empirical research studies being available that actively include migrant nurses. Many of the articles included in this review sre qualitative studies, that explore the lived experience of migrant nurses. The perspectives reported in these studies often describe ongoing discriminatory practices or exclusion. Examples of discrimination exist in each country for example Boese et al. (2013), reporting that migrant nurses are excluded from career progression pathways, Stuart (2012), reporting that migrant nurses often work as lower paid healthcare assistants, and Schiligen et al. (2019) reporting that migrant nurses experience more distress in their roles. These findings correspond to the theme of discrimination throughout the relevant literature. Despite the inclusion of the lived experience of some migrant nurses in the articles reviewed, this does not appear to translate into better experiences for migrant nurses. Future research that includes nurses with a migration background in the production of empirical data would address this gap in knowledge.

Structured integration educational programmes are highly valued by all stakeholders: therefore, it is all the more remarkable how infrequently they are addressed, scientifically substantiated and empirically investigated. Where such programmes are provided, they are reported to improve nurses’ health and well-being, retention, and patient safety. These programmes have specific requirements that differ from other educational initiatives and should be planned to meet the needs of those for whom they are provided. The two types of bridging courses are pre-registration courses or employment integration courses, each with specific pedagogical requirements but common themes of language and cultural integration. In Australia and the UK, the focus since the mid 1980s has been on pre-registration courses, with a language and clinical adaptation focus. The German governments Triple Win project is an employment integration programme for migrant nurses. This program offers support for nurses who migrate to Germany. The programme offers language support in nurses’ origin countries and professional orientation to nursing in Germany. The programme has resulted in 2,700 nurses being employed in Germany since 2013 but it is not transparent how nurse migrants are considered in this initiative (Deutsche Gesellschaft für Internationale Zusammenarbeit (GIZ) GmbH, 2021).

Among others, integration programmes should focus on the systematic development of language and communication skills for everyday and workplace situations. The ability to freely communicate in the first language of a destination country is given high value across all contexts by nurse migrants, healthcare representatives and service users. There is evidence for better outcomes for migrant nurses, patients and employers if language and communication support is integrated into educational programmes (Aggar et al., 2020; Holmes & Grech, 2015); therefore, policies should be guided by this evidence.

Successful integration schemes are typically multi-sector, with multiple stakeholders, e. g. local nurse managers, nurse educators, healthcare managers and representatives of educational institutions. This suggests that these programmes should have a strong focus on local institutional integration. Most importantly, including nurses with a migration background in the production of these programs and of empirical data, could address these issues.

The inclusion of nurses’ perspectives can be achieved directly by asking migrant nurses or, if that is not possible, by using relevant inclusive research. A broader evidence base, especially with regard to the views and experiences of migrant nurses and their educational support needs, should be promoted to make future immigration policy more needs-based, sustainable and ethically acceptable.

### Limitations

The REA methodology chosen for this work is not as comprehensive as a longer systematic review. However, the REA approach is a clear and transparent one that has helped to ensure that recognised standards of data production have been met.

Essentially, there would have been four ways to expand the findings presented here. First, the search criteria could have been expanded to include secondary research data and grey literature, including health policy documents and other data from government agencies. However, such data is extensive and would have required a different form of analysis, thus exceeding the scope of this project. It was decided, therefore, to include only empirical research actively involving migrant nurse in order to meet the demand for evidence-based migration policy measures in this area. In addition, further reviewers could have examined all extracted articles and not only those articles selected for inclusion in the final dataset. However, this process would possibly have led to further exclusion from the already small number of primary research articles.

Second, the research examined in this review does not provide in-depth information on the education and integration programmes, such as the breadth of the curriculum, the assessment procedures used, whether they give the participating nurse a transferable qualification such as a diploma or degree, or who is responsible for organising and commissioning the courses. This level of detail can be found in the literature outside the scope of this paper and may provide future research opportunities.

Third, the search strategy returned a small number of papers (n = 2) in the German context that were not available in the full text. The title was suggestive that these may be relevant to this review, however, further screening was not possible since these papers were over 19 years old.

Fourth, this review includes grey literature to understand the context of nurse migration, however, did not analyse this data in-depth. A vast amount of grey literature available from sources such as WHO, OECD and the International Monetary Fund yet much of this is not peer-reviewed and therefore did not meet the inclusion criteria. A broad overview of nurse migration by country was included in Appendix A to provide a brief statistical overview of nurse migration to further contextualise the discussions of articles included in this review.

This paper was prepared at the beginning of the COVID-19 pandemic and does not address resulting changes in global mobility. This emerging context will undoubtedly impact migration in ways that are not yet clear. The reduction in international travel will raise questions about countries’ reliance on nurse migration.

### Conclusion and Implications for Nursing Policy and Research

The findings of this paper suggest that healthcare research on nurse migration should be more inclusive of migrant nurses’ perspectives and experiences and incorporate these perspectives into policy and processes of nurse migration. Existing research has included the perspectives, knowledge, and experiences of migrant nurses in research, however, an opportunity exists to address the scale of this involvement to reflect the size of the deficits in nursing staff and the efforts of migrant nurses. There is also a clear opportunity to conduct larger research projects with migrant nurses beyond the relatively small studies found in this review; however, further research would require financial and structural initiatives by those invested in the process. We recommend further studies on the factors related to discrimination of migrant nurses. This research should look more comprehensively at the factors relating to discrimination and how research with and by migrant nurses can integrate into policy and practice.

We approached this project as researchers with a background in public health, nursing, education, and communication studies, but without a migration background ourselves. However, we wanted to understand and support the processes of nurse migration and create research based local policies that guide this process. The data suggests that such research should be integrated into national nurse recruitment strategies. Patient and Public Involvement (PPI) is an essential component of research practice therefore and should also be enacted in empirical research that supports policies around nurse migration. All research and policies should consider the ethical principles for nurse migration outlined by the WHO (2010, 2020a). Such integration is ethically mandated and is more successful for nurses and health systems socially and economically. The available research suggests that further development of migration strategies such as educational transition programmes, that consider or franchise migrant nurses, are urgently required. Further research could also examine these countries’ migration policy to determine if evidence exists to support that migrant nurses’ views and experiences were considered in policy design and identify how these policies are interpreted and executed in practice.

## Appendix A.

OECD data provide a sound basis for identifying some of the general differences and similarities between Australia, Germany, and the UK, and in particular for highlighting aspects relevant to nurse migration.

OECD data demonstrates close similarities in life expectancy and per capita healthcare expenditure in these three countries (OECD, 2020). What is striking, though, is the difference in the ratio of nurses to care personnel in these data. The UK reports a much higher ratio of care personnel to nurses than Germany and this value is not reported for Australia. This phenomenon may be explained by a different understanding of who is described and categorised as ‘Qualified Nurse’ and who as ‘Care Personnel’ in the respective statistics. Lauxen et al. (2019) discusses that the definition of nursing by a professional title may not be the same in every country and may be protected in law in different ways. In Germany, for example, there are three different professional titles for nurses that are mainly educated in a traditional hospital-based vocational training system: Adult Nurse (Gesundheits- und Krankenpfleger*in), Paediatric Nurse (Gesundheits- und Kinder­kranken­pfleger*in) and Care of the Older Person Nurse (Altenpfleger*in). However, none of them are registered by a professional body in Germany and only two meet the formal requirements of the European Union (EU) Directive for registered nurses in Europe. Due to different qualification standards, the title ‘Altenpfleger*in’ is not automatically recognised as a Qualified Nurse in other EU countries, which is why this professional group might be classified as ‘Care Personnel’ in other countries. In most other European countries, qualified nurses are graduated and registered, whereas care personnel undergo less demanding training. Such differentiation and educational aspects are important for the discourse on nurse migration, which is why they must be taken into account when comparing international statistics and research data.

Australia, Germany, and the UK have migrant nurses in their workforces, and these individuals are often recorded by country of origin (see Appendix A). These data show characteristics for the respective countries that should be considered when assessing the information available on nurse migration in research. First, the category of ‘migrant’ nurse is heterogeneous, but more so in Germany and the UK than Australia. The reported number of countries of origin or different nationalities varies widely: 12 in Australia, 16 in Germany and 129 in the UK (OECD, 2020, see Table 3 in Appendix A). These differences may be caused by the method of recording or level of detail of information recorded for migrant nurses in their destination countries. They may also demonstrate levels of diversity in migrant nursing workforces. As of 2017, 53.2% of overseas educated nurses working in Australia are from the UK. Sixty-four percent of recorded nurse migration to Australia is from countries where English is spoken as a first language by the majority of the population. Other countries, such as Germany, therefore, have a completely different starting situation due to the language barrier. This feature should be considered when discussing this cohort of research. Second, the number of migrant nurses recorded as ‘Others – not recorded’ is larger in Germany (29.8%) and the UK (26.7%) than in Australia (5.9%). Given the large amount of missing information and the broad characteristics of these data, further analysis may require additional research.

**Table 3.** OECD Health Workforce Migration Dashboard (Last year reported in full for all three countries, data last accessed June 2021). OECD Health Workforce Migration Dashboard (2020) available at https://stats.oecd.org/.

		*Numbers by country of nurse registration*		
		Australia	Germany	UK
*Nationality recorded*	UK	51.18
	India	14.452	407	15,726
	Phillippines	9.941		14,564
	New Zealand	6.915		197
	Others – not recorded	5.739	21,000	27,696
	Ireland	2.108		1,862
	South Africa	1.813		1,237
	China	1.397	106	290
	Zimbabwe	1.344		1,273
	United States	547		181
	Canada	539		121
	Malaysia	183		54
	Sri Lanka	86		151
	Poland		14,000	2,820
	Turkey		8,000	14
	Croatia		6,027	180
	Bosnia and Herzegovina		3,308	3
	Italy		3,285	4,609
	Romania		3,004	7,732
	Russia		2,854	11
	Serbia		2,334
	Greece		1,694	828
	Spain		1,548	6,493
	Portugal		1,464	4,955
	Morocco		925
	Tunisia		173	3
	Vietnam		153	1
	Others – Recorded from 105 countries (Range 1- 1,919)			12,421

## Appendix B.

**Table 4.** Data extraction table listing articles included in the review and a summary of their findings.

Author(s) and year	Title	Number of participants	Participant Characteristics	Research Design	Country	Synopsis and Outcome(s)
Aggar et al. (2020)	Experiences of internationally qualified registered nurses enrolled in a bridging program in Australia: A pilot Study	9	IQN enrolled in graduate nursing program	Mixed: questionairres and then focus groups	Australia	A transition to professional practice program may support the integration of IQNs into the Australian healthcare workforce.
Babatunde-Sowole et al. (2016)	“Coming to a Strange Land”.	20	West African nurses working in Australia	Qualitative interviews	Australia	The demand for and the importance of nurses and midwives in supporting migrant families is demonstrated by findings suggesting that social adjustment into the Australian culture has a significant impact on both the nuclear and extended family unit of women.
Boese et al. (2013).	Temporary migrant nurses in Australia: Sites and sources of precariousness	26	Nurses working temporarily in the Australian healthcare system	Qualitative interviews	Australia	Temporary migrant nurses are well integrated within the healthcare workforce in terms of formal wages and conditions, other stages in their migration pathways can be associated with precariousness
Brunero et al. (2008)	Expectations and experiences of recently recruited overseas qualified nurses in Australia	56	Nurses who arrived at hospital in the past 18 months	Quantitative	Australia	Nurses from culturally and linguistically diverse backgrounds reported not being employed in their chosen speciality and rating the utility of ward and hospital orientations more positively when compared to English speaking background nurses. Organisational and personalised approaches support the adjustment of OQNs into the nursing workforce.
Cecchin (1998)	International nurse education: implications for providers…Australian registered nurses who provide education programs for nurses from South East Asia	12	Nurse educators responisble for the education of Migrant nurses to Australia, from SE Asia	Qualitative	Australia	Cultural differences in education styles and culture exist. Specific preparation of providers of education programs for nurses from South East Asia was ad hoc and inadequate. Learning ‘on the job’ is more difficult for IQN's.
Crawford et al. (2016)	Are we on the same wavelength?’ International nurses and the process of confronting and adjusting to clinical communication in Australia	4	Culturally and Linguistically Different Nurses	Qualitative	Australia	Adjustment was identified as fundamental to the experiences of the RNs and this theme interrelated with each of the other themes that emerged: professional experiences with communication, ways of showing respect, displaying empathy, and vulnerability.
Deegan and Simkin (2010)	Expert to novice: experiences of professional adaptation reported by non-English speaking nurses in Australia	13	13 IQN'S from non-English speaking backgrounds and 3 nurse educators	Qualitative	Australia	Nurses reported feelings of disempowerment caused by discriminatory practices, professional isolation and unrealistic expectations by local nurses.
Holmes and Grech (2015)	CaLD nurses transition to Australian tertiary hospital practice: Exposing the reality – A mixed methods study	30	CaLD nurses.	Mixed	Australia	Without a hospital based orientation program participants were more likely to experience failure to ‘fit in’. This led to maladaptive behaviours where they did not actively seek, support or question practice through fear of drawing increased attention to themselves. Hospital orientation programs need to be developed as to the specific learning requirements of these nurses or risk exposing them to maladaptive behaviours and potential adverse events as a result of this learned behaviour.
Kishi et al. (2014)	A Model of Adaptation of Overseas Nurses: Exploring the Experiences of Japanese Nurses Working in Australia.	14	Japanese nurses working in Australia	Semi-structured interviews	Australia	The conceptual model of the adaptation processes of 14 Japanese nurses working in Australia includes the seeking, acclimatising, and settling phases.
Mapedzahama et al. (2012)	Black nurse in white space? Rethinking the in/visibility of race within the Australian nursing workplace	14	African nurses working in Australia	Qualitative interviews	Australia	Findings of nurse to nurse racism
Negin (2008)	Australia and New Zealand's contribution to Pacific Island health worker brain drain	Census data	652 Pacific Island born doctors and 3467 Australian nurses	Quantitative	Australia	Migration of Pacific Island health professionals to Australia and New Zealand is very high and contributes to the shortage of health workers in Pacific Island countries.
O’Callaghan et al. (2018)	Exploring the experiences of internationally and locally qualified nurses working in a culturally diverse environment	108	14% of hospital staff including OQN and AQN	Quantitative Survey	Australia	Adjustment to the Australian healthcare system for IQNs is challenging. There are a number of strategies that can support both IQNs in their integration, as well as all nurses to work more effectively together in a cross-cultural work environment.
Ohr et al. (2016)	The transition of overseas qualified nurses and midvices into the Australian healthcare workforce	65	OQN	Mixed	Australia	The support for the transition of overseas qualified staff into the Australian health care system continues to change, but it remains important to maintain support mechanisms and strategies to build the capacity and capability of the health workforce.
Omeri and Atkins (2002)	Lived experiences of immigrant nurses in New South Wales, Australia; searching for meaning	5	All from different countries. Only 1 from english speaking background	Qualitative	Australia	Social and cultural distance between nurses of the dominant culture and nurses from culturally and linguistically diverse backgrounds.
Philip et al. (2019)	Overseas Qualified Nurses’ (OQNs) perspectives and experiences of intraprofessional and nurse-patient communication through a Community of Practice lens	21	Non-English speaking overseas qualified nurses	Qualitative	Australia	OQNs can find communication in Australian healthcare complex and challenging making it an important area for learning.
Rumsey et al. (2016)	The consequences of English language testing for international health professionals and students: An Australian case study.	49	14 Health industry experts	Qualitative	Australia	This study has shown that some respondents have experienced difficulties in relation to the International English Language Testing System as part of their migration process. It was found that there is very little research into the effectiveness of the IELTS as it is currently administered for overseas health care professionals. Several recommendations are provided including areas for further research.
Salamonson et al. (2007)	Differences in universal diverse orientation among nursing students in Australia	816	Nursing students from Australia or CALD backgrounds	Quantitative	Australia	Nursing students from a non-English-speaking background could potentially enrich cross-cultural educational experiences for all students, but students who have recently settled in Australia may need support to feel a sense of connectedness.
Smith et al. (2011)	Rediscovering nursing: a study of overseas nurses working in Western Australia.	13	Migrant nurses working in Western Australia	Semi-structured interviews	Australia	Participants elaborated on some positive and some not-so-positive aspects of their experiences in their endeavour to integrate into the Western Australian metropolitan hospital setting
Takeno (2010)	Facilitating the transition of Asian nurses to work in Australia.	12	Korean and Japanese nurses working in Australia	Semi-structured interviews	Australia	Difficulties in adjusting to differences in the role of the nurse between Korea or Japan and Australia. This research highlights potential sources of misunderstanding and dissatisfaction which may be worth exploring in relation to other cultures.
Chun Tie et al. (2019)	Playing the game: a grounded theory study of the integration of internationally qualified nurses in the Australian healthcare system	217	Internationally and Australian Qualified Nurses	Qualitative	Australia	Supportive colleagues were critical to successful integration and retention of experienced nurses irrespective of where nurses obtain their nursing qualification. Additional orientation programs for international nurses could improve the experience of nurses migrating to work in Australia.
Timilsina et al. (2015)	Job satisfaction of overseas-qualified nurses working in Australian hospitals.	151	Overseas nurses working in five hospitals in Australia	Cross-sectional job satisfaction survey	Australia	Nurses from non-English-speaking backgrounds need to be supported early in their employment, especially with their communication skills.
Vafeas and Hendricks (2018)	A heuristic study of UK nurses’ migration to WA: Living the dream downunder	21	OQN from the UK now working in Australia	Qualitative	Australia	Feelings of belonging were necessary to make the move a success. Participants placed the need to develop new friendships and a replacement family as a high priority.
Walters (2008)	The experiences, challenges and rewards of nurses from South Asia in the process of entering the Australian nursing system	15	New OSN from non English speaking backgrounds	Qualitative	Australia	The main themes identified were Trust and fear; English language requirements; Immigration issues; Belonging, integration and family and Living and working in the West.
Zhou et al. (2011)	The concept of difference and the experience of China-educated nurses working in Australia: A symbolic interactionist exploration.	28	China-educated nurses working in the Australian health care system	Qualitative interviews	Australia	Negative meanings were ascribed to difference which in turn legitimised inequality and held the potential to perpetuate racism.
Lauxenet al. (2019)	Pflegefachkräfte aus dem Ausland und ihr Beitrag zur Fachkräftesicherung in Deutschland. Das Fallbeispiel Hessen	40	16 foreign nurses and 24 local nurses.	Mixed	Germany	The results indicate that the potential of nursing immigration for securing skilled workers has increased, especially in health and nursing or in the hospital sector. In contrast, the potential still appears low in nursing for the elderly and children.
Peters and Braeske (2016)	Pflegekrafte aus Vietnam: Erste Erfahrungen der deutschen Altenpflege.	100	Vietnamese nurses working in Germany	Semi-structured interviews	Germany	Professional, cultural and linguistic support are useful for successful migration.
Schilgen et al. (2020)	The Extent of Psychosocial Distress among Immigrant and Non-Immigrant Homecare Nurses—A Comparative cross Sectional Survey	249 and 287	The Extent of Psychosocial Distress among Immigrant and Non-Immigrant Homecare Nurses—A Comparative cross Sectional Survey	Quantitative	Germany	There was no significant difference in the extent of psychosocial distress experienced by immigrant and non-immigrant homecare nurses. Somatic symptom burdens most strongly predicted nurses’ psychosocial distress, in general. A functioning relationship with colleagues and superiors improved immigrant nurses’ psychosocial distress, while shift work arrangements benefitted non-immigrant nurses.
Schilgen et al. (2019)	Work-related barriers and resources of migrant and autochthonous homecare nurses in Germany: A qualitative comparative study	48	24 migrant and 24 ‘autocthonous’ nurses employed in the german homecare sector	Qualitative	Germany	Migrant and first country nurses share similar coping strategies to master occupational burdens. Differences in language is a main stressor, which impedes a functioning team collaboration as well as a working nurse-client relationship. More autochthonous than migrant and minority nurses report these differences as stressful. Migrant nurses of different origin perceive their status as migrants as a sense of community by sharing the same destiny – this appears as an important resource for migrant and minority nurses.
Kumpf et al. (2016)	Erfahrungen ausländisher Pflegekräfte in Deutschland	10	5 foreign nurses and 5 educated in Germany	Qualitative - structured interviews	Germany	The main challenges identified were language barriers, socio-cultural conflicts and perceived discrimination.
Aboderin (2007)	Contexts, motives and experiences of Nigerian overseas nurses: understanding links to globalisation.	32	25 Nigerian nurses working in the UK, and 7 returnee nurses to Nigeria	Qualitative interviews	UK	Nurses’ migration motives arise from a deterioration in their economic, work and status situation over the past two decades in the context of a macro-economic decline in Nigeria. Their decisive motivation is to gain financially with a view to achieving certain material standards and prospects for self and children in Nigeria. Contrary to their expectations, they experience a loss in professional and social status in the UK.
Adhikari (2013)	Empowered Wives and Frustrated Husbands: Nursing, Gender and Migrant Nepali in the UK	9	Nepalese Nurses working in the UK	Qualitative	UK	International nurse migration affects nurses’ immediate family dynamics. This article illustrates how migrant nurses’ husbands accept a ‘compromised’ social position, from being family bread-winners in Nepal to dependent husbands in the UK.
Adhikari and Grigulis (2014)	Through the back door: nurse migration to the UK from Malawi and Nepal, a policy critique.	168	46 Malawian nurses and 122 Nepalese nurses	Qualitative interviews	UK	Shifting forces of nursing workforce demand and supply, leading to abrupt policy changes, have significant implications on overseas nurses’ lives, and can leave nurses ‘trapped’ in the UK.
Adhikari and Melia (2015)	The (mis)management of nurses in the UK: a sociological study	21	Nepalese Nurses working in the UK	Ethnopgraphic study involving 21 in-depth interviews	UK	Nepali migrant nurses are highly qualified and experienced in specialised areas such as critical care, management and education. However, these nurses end up working in the long-term care sector, providing personal care for elderly people - an area commonly described by migrant nurses as British Bottom Care (BBC). This means that migrant nurses lack career choices and professional development opportunities, causing them frustration and lack of job satisfaction.
Al-Hamdan et al. (2015)	Experiencing transformation: the case of Jordanian nurse immigrating to the UK	25	Jordanian migrant nurses working in the UK	Qualitative	UK	Social transformation is an integral and inseparable part of engagement with professional organisation(s) in the host community. This has direct implications for clinical practice and patient safety.
Alexis (2009)	Overseas trained nurses’ perception of UK nurses’ caring attitudes: a qualitative study.	12	Migrant nurses working in the UK	Semi-structured interviews	UK	This paper concludes by indicating that teamwork, being empathetic, understanding and reducing emotional labour for overseas nurses could lead to a more satisfied working environment for overseas nurses in the NHS in the UK.
Alexis (2013)	Internationally educated nurses’ experiences in a hospital in England: an exploratory study.	12	Migrant nurses working in the UK	Semi-structured interviews and focus groups	UK	Internationally educated nurses encountered a number of challenges to their working practices in an English hospital, and there is a need for both IENs and home-grown nurses to adapt to each other cultural differences.
Alexis and Vydelingum (2009)	Experiences in the UK National Health Service: The overseas nurses’ workforce	188	81.9%female and 18.1% male, the age group 21-40 and over with different grades of knowledge	Quantitative	UK	Equal opportunity as well as opportunities for skills development and training should be universal within the NHS as this could improve the inequitable treatment of migrant nurses that is apparent throughout the UK.
Alexis et al. (2007)	Engaging with a new reality: experiences of overseas minority ethnic nurses in the NHS.	6	Migrant nurses working in the UK	In-depth interviews	UK	There is a need for overseas nurses to be treated fairly and with respect particularly in the light of an acute labour shortage of nurses in the NHS. The findings suggest that overseas minority ethnic nurses’ experiences have been mixed, with some positive as well as negative experiences, within a process that devalues them as workers.
Alexis and Shillingford (2015)	Internationally recruited neonatal nurses’ experiences in the National Health Service in London	13	Qualitative interviews	Qualitative	UK	Overseas nurses experienced some challenges to their working lives; however, good preparation is important when recruiting them to work in the NHS.
Allan (2010)	Mentoring overseas nurses: barriers to effective and non-discriminatory mentoring practices	130	Interviews were undertaken with 93 overseas nurses and 24 national and 13 local managers and mentors from six research sites	Structured interviews	UK	Overseas nurses are discriminated against in their learning by poor mentoring practices; equally, from these data, it appears that mentors are ill-equipped by existing mentor preparation programmes to mentor overseas-trained nurses from culturally diverse backgrounds.
Allan and Larsen (2003)	Learning from others: overseas nurses’ views of UK nursing.	67	Migrant nurses working in the UK	11 focus groups	UK	Migrant nurses identified as highly skilled.
Allan and Westwood (2016)	Internationally Educated Nurses Working as Healthcare Assistants in the UK: Perceived barriers to UK nurse registration among non EU/EEA nurses	11	Overseas trained nurses working as Healthcare Assistants	Qualitative	UK	Intergration programmes to support migration are essential. There is also a need for increased monitoring and research activities in order to be able to describe, classify and design the migration process in more detail and to promote internal activities for sustainable integration.
Allan et al. (2009)	Overseas nurses’ experiences of discrimination: a case of racist bullying?	3	Migrant nurses working in the UK	Case studies	UK	Racist bullying is specifically identified as a form of bullying
Alonso-Garbayo and Maben (2009)	Internationally recruited nurses from India and the Philippines in the United Kingdom: the decision to emigrate.	21	6 Indian and 15 Phillipino nurses worling in the UK	Cross-sectional interviews	UK	Migration is often motivated by financial implications. Professional status and breadth of clinical opportunities can also influence migration.
Daniel et al. (2001)	Expectations and experiences of newly recruited Filipino nurses	-	Fillipino nurses working in London	Focus groups	UK	Adjusting to the new system of health care proved stressful but was helped by the provision of support services. Factors that may promote successful adaptation and retention included equal opportunities with respect to training and promotion and the use of culturally sensitive orientation programmes.
Hardill and Macdonald (2010)	Skilled International Migration: The Experience of Nurses in the UK	16	16 Semi structured interviews	Qualitative	UK	Most nurses migrated as solos, experienced downward occupational mobility, and are used as a temporary stop-gap to fill labour shortages.
Larsen (2007)	Embodiment of discrimination and overseas nurses’ career progression.	2	Overseas nurses working in the UK	In-depth interviews	UK	Social and institutionalised discrimination in the UK healthcare sector may be internalised by overseas workers and affects their professional careers
Leone et al. (2020)	Experience of mobile nursing workforce from Portugal to the NHS in UK: influence of institutions and actors at the system, organization and individual levels.	27	Portugese nurses working in the UK	Semi-structured interviews	UK	Migration is accomplished through constant interaction between institutions and individual actors at different levels. Understanding the influencing factors as well as the complex and dynamic nature of a professional's decision-making can design more effective retention responses
Henry (2007)	Institutionalised disadvantage: older Ghanaian nurses’ and midwives’ reflections on career progression and stagnation in the NHS.	22	Ghanain nurses’ and midwives working in the NHS	Semi-structured interviews	UK	Many Ghanaian nurses and midwives can experience difficulty in progressing into senior positions because of cultural differences and gaps in knowledge.
Likupe (2015)	Experiences of African nurses and the perception of their managers in the NHS.	15	African nurses working in the NHS	Semi-structured interviews	UK	Racism and discrimination towards black and ethnic minority nurses are present in the National Health Service despite equality Acts
O’Brien and Ackroyd (2012).	Understanding the recruitment and retention of overseas nurses: realist case study research in National Health Service Hospitals in the UK.	Seven cohorts of overseas nurses	Observational cohort data and semi-structured interviews	Qualitative	UK	Successful assimilation is often hindered by the presence of occupational closure mechanisms, by which home nurses effectively excluded recruits from participation and promotion
Okougha and Tilki (2010)	Experience of overseas nurses: the potential for misunderstanding.	12	Nurses from Ghana and the Phillipines working in the UK	Structured interviews	UK	They argue for a shift of focus from the newcomers and highlight the importance of preparing mentors and colleagues to facilitate effective adaptation through cultural awareness, knowledge and sensitivit
Sedgwick and Garner (2017)	How appropriate are the English language test requirements for non-UK-trained nurses? A qualitative study of spoken communication in UK hospitals	4 + 11	Tracing study with 4 nurses then 11 for focus groups	Qualitative	UK	In addition to linguistic knowledge and fluency, nursing requires considerable cultural and pragmatic knowledge and competence.There is an argument for including socio-pragmatic competence in language tests specifically designed for nurses
Stuart (2012)	Overseas nurses’ experience as support workers in the UK	48	Overseas nurses working as suppprt workers	Qualitative interviews and observation	UK	Support workers from overseas may have prior knowledge and experience that is not recognised in their current workplace. They can witness care and practice they know is wrong but lack the skills to know not what to do Support workers ask to be valued and trusted. They are a vulnerable group and can experience bullying by peers and supervisors.
Stubbs (2017)	Recruitment of nurses from India and their experiences of an Overseas Nurses Program	16	Longitudinal and serial interviews	Qualitative	UK	Autonomy disparity, language barriers and cultural differences need to be recognised and acknowledged by multi-disciplinary teams, by allowing sufficient time and additional support for non-English nurses undergoing overseas nursing programs.
Terry et al. (2013)	The Effect of Fluency in English on the Continuing Professional Development of Nurses Educated Overseas	18	Six nurse educators, six native english speaking nurses and six non-native english speaking nurses	Structured interviews	UK	Fluency in academic nursing English is necessary for successful continuing professional development. Educators should use and develop strategies to encourage integration in the classroom between nurses who were educated in the United Kingdom and those who were educated overseas and are not native English speakers to support critical thinking and engagement by all participants.
		*Qualitative*	*Quantitative*	*Mixed*	*Total*	
	Australia	17	5	3	0	
	UK	27	0	0	0	
	Germany	3	1	1	0	

*Note*. AQN = Australian Qualified Nurse; IELTS = International English Language Testing System; IEN = Internationally Educated Nurses; OSN = Overseas Nurses; RN = Registered Nurse.
